# Headache as a Presenting Symptom of Deep Brain Stimulator Generator Failure

**DOI:** 10.7759/cureus.16726

**Published:** 2021-07-29

**Authors:** Trevor Luck, Dorian M Kusyk, Donald Whiting

**Affiliations:** 1 Department of Neurological Surgery, Drexel University College of Medicine, Philadelphia, USA; 2 Department of Neurological Surgery, Allegheny Health Network, Pittsburgh, USA

**Keywords:** neuromodulation, obesity, lateral hypothalamic area, mammilotegmental fascisculus, dbs, deep brain stimulation

## Abstract

While a headache can have a wide variety of clinical presentations, it may occasionally be a red flag for underlying pathology that should prompt further investigation. Here, we present a case report demonstrating headache as an uncommon symptom of deep brain stimulation (DBS) device failure and discuss its clinical significance in the rapidly expanding list of current indications of DBS treatment. A 61-year-old female underwent bilateral hypothalamic DBS implantation for refractory morbid obesity. After a successful course involving significant weight loss, the patient began to experience worsening of her chronic headaches, refractory to her existing regiment. On interrogation, her generator was found to be depleted and its subsequent replacement led to a near total resolution of her headaches. This represents one of the few reported instances of headache as a sign of device failure in DBS treatment, thus adding to the wide possibility of headache presentations and their underlying pathology.

## Introduction

Over the past decade, deep brain stimulation (DBS) treatment has rapidly expanded its potential use to 11 current indications, progressing beyond the traditional host of movement disorders such as Parkinson’s disease to diagnoses such as depression, addiction, cluster headache, and morbid obesity [1]. Each of these indications has a target of interest: for Parkinson’s, it has historically been basal ganglia elements that receive dopaminergic input such as the globus palladus internus and subthalamic nucleus; for obesity, it is the lateral hypothalamus, a key regulatory center for satiety [1]. One feature that sets this new generation of DBS patients apart from their movement disorder peers is the lack of overt motor symptoms such as tremors that might immediately respond to treatment as well as promptly reflect device malfunction. As a result, these newer indications present new challenges in device management, making device troubleshooting more difficult and device failure easier to miss.

Historically, the return of a patient’s symptoms was enough to bring a patient to clinic for evaluation of their generator. While this might hold true for movement disorders and painful conditions such as cluster headache, as more amorphous conditions are treated with neuromodulation, this paradigm will no longer suffice to clinically suspect device failure for all DBS patients [2]. Since device failure is one of the most common problems facing DBS patients [3], it is imperative to better characterize the presenting symptomology of battery depletion in these non-movement disorder DBS patients.

Although headache would be an obvious sign of battery depletion in treatment for cluster headache, it is not intuitive or expected in other indications for DBS [4]. This case report highlights headache as a symptom of device failure in the setting of DBS treatment for obesity and highlights the importance of suspecting battery depletion when new or atypical symptomology presents in the growing population of non-movement disorder DBS patients.

## Case presentation

**Figure 1:**
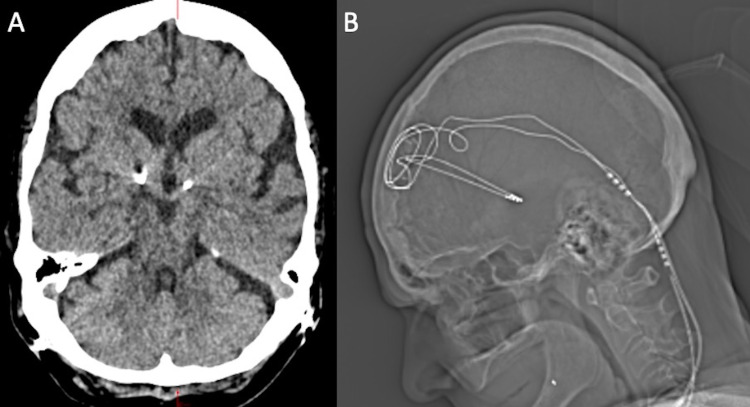
(A) Axial postoperative head CT demonstrating bilateral hypothalamic lead placement. (B) Lateral postoperative head x-ray demonstrating bilateral hypothalamic lead placement.

This is a 61-year-old female with a history of morbid obesity refractory to medical management and conventional surgery, now successfully treated with bilateral hypothalamic DBS for the past 12 years. She was initially implanted in 2008 according to previously described protocols targeting the lateral hypothalamic area (LHA) for obesity (Figures 1A, 1B) [5]. Prior to implantation, the patient weighed approximately 408lbs and has successfully proceeded to lose over 200lbs reaching a nadir of 197lbs. During her treatment, she had undergone one previous generator replacement in 2014. In 2020, the patient presented to the clinic with four months of progressively worsening headaches. She did endorse a long-term history of migrainoid headaches starting from adolescence. She described them as “day to day” in frequency, 3/10 in severity, and starting at the top of her head and radiating to her neck. These headaches were historically controlled by a regiment of amitriptyline, gabapentin, and acetaminophen by her neurologist. However, for four months, she noted worsening severity in her daily headaches, described as 8/10, and uncontrolled on her usual regimen. She experienced no significant changes in weight or appetite during this time. On our evaluation, she was found to have depleted generators and underwent replacement. Interestingly, on follow-up six weeks later, she reported a near-complete resolution of her headaches, down to her baseline of 3/10. She is now six months out from generator replacement, reporting that she has continued improvement in her symptoms and that her neurologist is weaning down her amitriptyline due to the improvement. She gave written informed consent to be described in this case report.

## Discussion

The issue of battery depletion in DBS patients is a well-known phenomenon, and any return of symptoms will often bring patients into the clinic for investigation of their generators. These symptoms can range from very clear signs such as a return of a tremor or dystonia in movement disorders, headache in cluster headache, and neuropsychiatric signs in patients with devices implanted for OCD [6,7]. More serious symptoms are also possible - in recent years, particular focus has been drawn to a neuroleptic-like malignant syndrome in certain Parkinson's disease patients on device failure and status dystonicus in dystonia patients [8].

The challenge of timing battery replacements is further compounded by inaccuracies in generator life prediction. While many patients are given estimates for the life of their device, research has repeatedly demonstrated that these ranges are ambiguous and often incorrect [9]. More accurate calculations of battery life are made more complex by patient-specific factors, electrode impedance, changing programming parameters, or even turning generators off at night [10]. Though most models now come with “Elective Replacement Indicator” messages on patient programmers, this feature has not removed that need for battery life estimators which take into account generator settings and patient symptoms [2]. As neuromodulation expands to new pathologies, it is vital that more work is done to help identify new signs and symptoms that may accompany generator depletion.

To our knowledge, this case represents the first report of headache as a presenting sign of DBS device failure in the setting of obesity. Although it is not immediately obvious why headaches arose as a result of hypothalamic DBS failure, one possible explanation could stem from our current understanding of DBS treatment for cluster headaches [11,12]. In the case of DBS treatment for cluster headache, there is debate about whether the effect of DBS modulation on cluster headache is due to focal modulation of brain nuclei such as the posterior hypothalamic area (PHA), or modulation of tracts and their downstream targets such as the mammilotegmental fasciculus (MTF), as reported by Fernandez et al. [12]. While the PHA is about 8 millimeters away from the LHA along the anteroposterior axis and its involvement may be unlikely, the course of the MTF runs in close proximity to the LHA and it is not unreasonable to think that some off-target stimulation could be implicated in the worsening presentation and subsequent resolution of this patient’s headaches.

Given the patient’s longstanding history of migrainoid headaches, it may be possible that some sort of tolerance or dependence may have developed over the 12 years of treatment our patient underwent, and that sudden withdrawal of neuromodulation due to battery failure triggered worsening of her existing headaches through these same pathways. This would be consistent with the notion that a neuroplastic mechanism is involved in DBS treatment for cluster headache, given that most patients reach a peak in therapeutic response months after device implantation rather than immediately [13]. Whatever the mechanism may be, this case highlights the importance of having a low threshold to clinically suspect device malfunction in this new horizon of DBS indications.

## Conclusions

This case highlights that device failure should be high in a clinician’s suspicion when evaluating novel symptoms in a DBS patient, even when it may not be intuitively linked to the underlying treatment indication. With the growing indications for DBS beyond movement disorders, it is important to better detect the more subtle symptoms of device failure. Further research is needed to help identify other possible signs and symptoms of failure, and whether these symptoms are target-specific or not.

## References

[1] Lee DJ, Lozano CS, Dallapiazza RF, Lozano AM (2019). Current and future directions of deep brain stimulation for neurological and psychiatric disorders. J Neurosurg.

[2] Fakhar K, Hastings E, Butson CR, Foote KD, Zeilman P, Okun MS (2013). Management of deep brain stimulator battery failure: battery estimators, charge density, and importance of clinical symptoms. PLoS One.

[3] Jitkritsadakul O, Bhidayasiri R, Kalia SK, Hodaie M, Lozano AM, Fasano A (2017). Systematic review of hardware-related complications of deep brain stimulation: do new indications pose an increased risk?. Brain Stimul.

[4] Aibar-Durán JÁ, Holzapfel MJA, Rodríguez RR, Nieto RB, Arnall CR, Teixido JM (2020). Occipital nerve stimulation and deep brain stimulation for refractory cluster headache: a prospective analysis of efficacy over time [PREPRINT]. J Neurosurg.

[5] Whiting AC, Sutton EF, Walker CT (2019). Deep brain stimulation of the hypothalamus leads to increased metabolic rate in refractory obesity. World Neurosurg.

[6] Vora AK, Ward H, Foote KD, Goodman WK, Okun MS (2012). Rebound symptoms following battery depletion in the NIH OCD DBS cohort: clinical and reimbursement issues. Brain Stimul.

[7] Miocinovic S, Ostrem JL, Okun MS, Bullinger KL, Riva-Posse P, Gross RE, Buetefisch CM (2020). Recommendations for deep brain stimulation device management during a pandemic. J Parkinsons Dis.

[8] Sauer T, Wolf ME, Blahak C, Capelle HH, Krauss JK (2017). Neuroleptic-like malignant syndrome after battery depletion in a patient with deep brain stimulation for secondary parkinsonism. Mov Disord Clin Pract.

[9] Okun MS, Tagliati M, Pourfar M, Fernandez HH, Rodriguez RL, Alterman RL, Foote KD (2005). Management of referred deep brain stimulation failures: a retrospective analysis from 2 movement disorders centers. Arch Neurol.

[10] Montuno MA, Kohner AB, Foote KD, Okun MS (2013). An algorithm for management of deep brain stimulation battery replacements: devising a web-based battery estimator and clinical symptom approach. Neuromodulation.

[11] Leone M, Cecchini AP (2016). Deep brain stimulation in headache. Cephalalgia.

[12] Seijo-Fernandez F, Saiz A, Santamarta E (2018). Long-term results of deep brain stimulation of the mamillotegmental fasciculus in chronic cluster headache. Stereotact Funct Neurosurg.

[13] Pedersen JL, Barloese M, Jensen RH (2013). Neurostimulation in cluster headache: a review of current progress. Cephalalgia.

